# Inhibition of α-synuclein aggregation by hesperidin as a potent anti-amyloidogenic polyphenol: A computational approach and MM-PBSA /ADMET analysis

**DOI:** 10.1016/j.bbrep.2025.102318

**Published:** 2025-10-29

**Authors:** Zainab Abdullah Waheed, Haider Khabt Aboud, Jasem Hanoon Hashim Al-Awadi, Azhaar Mousa Jaffar Al-Mousawy, khudhair Rashid khudhair Alzubaidi

**Affiliations:** a Department of Medical Laboratory Techniques, Institute of Medical Technology-Al Mansour, Middle Technical University, Baghdad, Iraq; b Department of Chemistry, College of Science, University of Babylon, Babylon, Iraq; c Department of Biology, College of Science, University of Kerbala, Karbala, Iraq; d Department Medical Laboratory Techniques, University of Al-Furat Al-Awsat Technical, Karbala, Iraq; e Department of Animal Biology, Faculty of Natural Sciences, University of Tabriz, Tabriz, Iran

**Keywords:** α-Syn, Parkinson's disease, Aggregation, MD simulation, Flavonoid compounds, Hesperidin

## Abstract

A significant part of amyloidogenic illnesses is played by protein misfolding and aggregation caused by intrinsically disordered protein (IDP) self-assembly in Parkinson's disease (PD). In PD, cytotoxic amyloid aggregates of aberrant alpha-synuclein (α-syn) are formed in motor neurons, causing neurodegeneration. Beta-sheet-rich amyloid aggregates are a promising target for mitigating their neurodegenerative consequences. A significant amount of work has been invested in developing chemical compounds that either prevent aggregates from forming or facilitate their breakdown. Finding them might provide a workable strategy for creating a powerful remedy. Several studies indicate that neurological disorders can be treated using small-molecule inhibitors derived from polyphenolic flavonoid compounds. We have thus identified a potential flavonoid molecule that can effectively inhibit the amyloidogenic activity of α-syn through molecular docking and molecular dynamics (MD) simulations. Hesperidin, Morin, and Myricetin were shown to be potential therapeutic leads in the initial screening of flavonoids. Compared to other compounds, the hesperidin-α-Syn combination showed a larger residual energy contribution (ΔE binding −92.69 ± 0.31 kJ mol^−1^) and significant binding (6.58 kcal/mol). Secondary structural analysis showed an increased tendency for β-sheets (35 %), with the percentage of β-sheets decreasing in the presence of Hesperidin (29 %). The results showed that Hesperidin binding resulted in a significant reduction in hydrogen bonding between α-syn peptide chains compared to unbound protein. The findings demonstrated that, in contrast to other compounds, Hesperidin alters protein flexibility, hydrophobicity, and structural stability. Therefore, Hesperidin may be a promising treatment option to reduce the negative consequences of PD by efficiently mitigating α-syn amyloid-induced neurotoxicity.

## Highlights

• Hesperidin binds well to α-synuclein and prevents its aggregation.
• Hesperidin can cause structural changes.
• Hesperidin could serve as a potential treatment for intractable PD affecting humans.

## Graphical abstract

**Figure:**
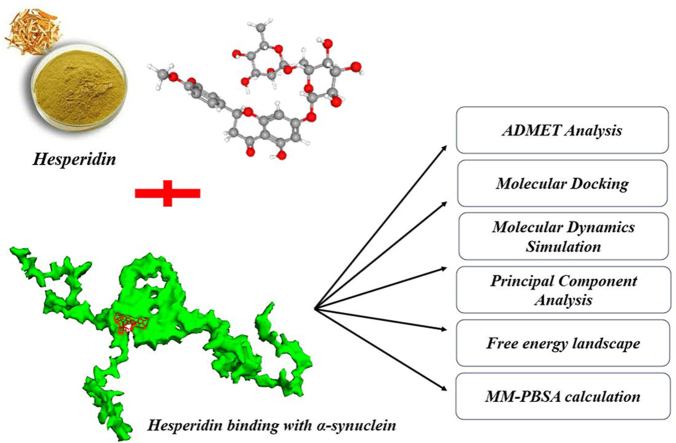

## 1 Introduction

Neurodegenerative conditions (NDs) such as Parkinson's disease (PD) are more or less derived from the formation of aberrant protein oligomers that are cytotoxic in nature [1]. One of the key features of these diseases is the deposition of protein aggregates, more so high β-sheet content structures, which are involved in a cascade of toxic cellular pathways. In PD specifically, α-synuclein (α-syn) oligomers have been identified as the key pathogenic players. These oligomers pile up inside cellular inclusions called Lewy bodies and Lewy neurites and cause the death of dopamine-secreting neurons of the substantia nigra [2].

α-Synuclein protein is a 140-amino-acid protein that has neither tryptophan nor cysteine residues. It is typically topologically segregated into three regions: the N-terminal domain (residues 1–60), which is lipid-binding and helical in structure; the central domain (residues 61–95), which is hydrophobic and facilitates self-association and fibril formation through β-sheet stacking; and the acidic C-terminal domain (residues 96–140), which contains dense aspartic and glutamic acid and is responsible for inhibiting fibril rapid growth [3]. Since it is a naturally disordered protein, α-synuclein can convert into amyloid fibril structures. α-Synuclein has also been found to occur in multiple structural conformations. Conversion of monomeric units into oligomers and then into fibrils has been presumed to be the initial step in pathological process in disease development [4]. A number of internal and external factors have been proven in the laboratory to affect α-synuclein aggregation. These include differences in temperature, pH, certain point mutations [5], N-terminal acetylation [6], and exposure to organic solvents, metal ions, and salts [7]. Even after extensive studies, the precise mechanisms in the process of aggregation are yet to be understood. Moreover, the physiological role of α-synuclein is still not fully understood. It is speculated to be implicated in cognitive processes, regulate dopamine neurotransmitter release [8], and help regulate synaptic vesicle dynamics [9]. After α-synuclein has become aggregated, however, it transforms from its monomeric structure to ordered amyloid fibrils, which cause neuronal death. These fibrils consist of several protofilaments whose β-sheets are perpendicular to the fibril axis [4,10,11]. Thus, treatments that block or suppress pathologic assembly of α-synuclein amyloid fibrils may prevent neuronal damage.

Despite our deepened knowledge of Parkinson's disease (PD) and other protein misfolding diseases, and many experiments showing that peptides and small molecules are effective to stop protein aggregation in vitro, no treatment is yet effective in preventing the deposition or formation of toxic oligomers [12]. Therefore, one of the most encouraging treatments for amyloid-caging diseases is to inhibit protein misfolding and stop amyloid fibril formation. Several strategies have been proposed that may be used for this purpose, including (i) fixing amyloid-prone peptides' and proteins' native conformation; (ii) promoting clearance of misfolded intermediates; and (iii) blocking the process of self-assembly or re-routing amyloid formation onto harmless pathways [13,14]. Due to this need, there has been rigorous screening of anti-amyloid activity candidates. Among many classes of small molecules, and peptides which have been investigated for research, the phytochemicals have been of interest due to their potential role in PD, and other proteinopathies. These endogenously present substances, ubiquitously present in both food plants, as well as herbs [15], are exemplified by polyphenolic flavonoids, which may be particularly emphasized for their antioxidant activity, and its ability to interfere with various steps of the amyloid forming pathway. Amyloidogenesis has been shown to be induced by neurotoxic stress [16]. Due to their functionally distinctive moieties, these molecules are able to bind to numerous biological targets, i.e., proteins, and induce conformational changes through changing the way proteins interact with other proteins [17]. Along with their antioxidant [18], antimicrobial [19], and antitumor [20] activity, polyphenolic flavonoids have been extensively shown to interact with amyloid-forming peptides and proteins, change or reverse their process of aggregation, and significantly reduce their resulting toxicity [21]. Past studies have shown that polyphenols and other low-molecular-weight compounds can disrupt the formation of beta-sheet structures—key factors in amyloid deposition in neurological disorders such as Parkinson's [12]. In addition, flavonoids were also shown to be protective against amyloid buildup in lethal conditions like Alzheimer's disease and amyotrophic lateral sclerosis (ALS) associated with SOD1 mutations [[22], [23], [24]]. On this backdrop, we centered our research on investigating how polyphenolic flavonoids disrupt α-synuclein aggregation. The development of powerful computational chemistry has made it possible to model accurately the structures of biomolecules, and thus scientists can explore the complex binding mechanisms of candidate drugs with proteins. Computational drug discovery can accelerate the challenging process of new drug design and optimization. The impact of computational structure-based drug design (SBDD) on drug discovery has intensified in the past decade due to the rapid development of faster architectures and better algorithms for high-level computations in a cost-effective manner. Classical molecular dynamics (MD) simulations today allow the implementation of SBDD strategies that fully account for the structural flexibility of the overall drug-target model system. The main advantage of MD is the explicit treatment of structural flexibility and entropic effects. This allows for more accurate estimation of the thermodynamics and kinetics associated with drug-target recognition and binding, as better algorithms and hardware architectures increase their use. MD simulations can help understand and use competitive inhibition mechanisms for drug design and investigate the thermodynamic properties of protein binding sites. Another key challenge in drug discovery stems from the fact that potency, although essential, is only the first step towards discovering a promising drug candidate. Once a potent inhibitor is discovered, it must be tuned into a drug compound with a desirable ADMET profile in the lead optimization phase. Most often, this is a very key step and a real bottleneck in drug discovery. The ability to predict thermodynamics and binding kinetics through MD-based methods has the potential to also impact lead optimization, indicating which compounds and modifications are most desirable, as previously reported [25]. Prospective studies (experimental validation) will serve as the final proof of concept to demonstrate that MD can indeed be used to aid in the costly and very challenging process of drug discovery. Thus, we utilized molecular docking and MD simulations in activity prediction and therapeutic potency of nature-derived anti-aggregation drugs against α-synuclein.

## 2 Computational methods

### 2.1 Structural optimization

The three-dimensional α-synuclein structure in solid-state NMR (PDB ID: 2N0A) was retrieved from the Protein Data Bank (PDB) [26]. The GROMACS software package was employed to optimize and refine the dimeric protein structure [27].

### 2.2 Ligand development

Chemical conformations of flavonoid molecules, as ligands, were retrieved from PubChem database in SDF format. Molecular geometries were subsequently geometrically optimized using MOPAC with the semi-empirical AM1 Hamiltonian method for the optimal conformations [28]. The optimized ligand conformers were then utilized in molecular docking experiments.

### 2.3 Molecular docking studies

AutoDock version 4.2.3 was utilized to assess binding interactions between α-synuclein and the synthesized ligands. This procedure utilized pre-computed energy grids and an optimized search algorithm for this specific purpose to identify the optimum binding conformations. In the simulation model, the α-synuclein protein was fixed, while torsion flexibility was permitted for ligand molecules so that they may adopt best orientations during docking. Aggregation-prone binding sites were chosen as the ligand-binding site because they play an essential role during the protein aggregation process [29]. Partial charges at atomic level were set with Kollman parameters on the protein and Gasteiger's method on the ligands. Docking was executed by the Lamarckian genetic algorithm by combining the semi-empirical energy predictions and the grid-based maps obtained using AutoGrid with default parameters. Grid spacing of 0.375 Å was utilized in order to accommodate important residues on the binding interface. The aggregate binding energy for every complex in the form of torsional, internal, and interaction energy terms was calculated. Optimal energetics and binding pose ligand-protein pairs were further examined and visualized with PoseView.

### 2.4 Molecular dynamics simulations

MD simulations were conducted using GROMACS 2022.1. LigParGen web server (https://traken.chem.yale.edu/ligpargen/) with CHARMM-compatible parameters generated ligand topology files. The ligand and protein parts were simulated under the CHARMM27 force field. Complex structures were placed in a dodecahedral water box with 10 Å buffer with the TIP4P water model. 35 sodium ions (Na^+^) and 27 chloride ions (Cl^−^) were added to obtain physiological salt concentration (0.15 mM) to provide overall charge neutrality. The initial structures were then relaxed using energy minimization with the steepest descent algorithm for 10,000 steps. This was then followed by system equilibration for 10 ns in an NVT ensemble at a temperature of 310 K, with the number of particles, volume, and temperature being constant. Before this step, the systems were subjected to an unconstrained 250-ns MD simulation in NPT conditions (constant number of particles, 1 atm pressure, and 310 K temperature). Constraints were imposed on the bonds so that the lengths of covalent bonds were kept constant using the LINCS algorithm. Simulations were performed with a timestep of 1 fs and cutoff values of 1.2 nm for both Coulombic and Lennard-Jones forces. The Particle Mesh Ewald (PME) approach was used to handle long-range electrostatic interactions.

Important dynamic and structure analysis—root mean square deviation (RMSD), root mean square fluctuation (RMSF), hydrogen bonding, radius of gyration (Rg), solvent-accessible surface area (SASA), and secondary structure profiles using DSSP—were carried out as mentioned earlier [[30], [31], [32], [33]]. The outcome shows the average of all the parameters and analysis. Moreover, Principal Component Analysis (PCA) and free energy landscape (FEL) were conducted with gmx covar and gmx anaeig [34], which diagonalized the covariance matrix to produce projections along principal components. Furthermore, the binding energy of the ligands to the protein, were analyzed using the MMPBSA (Molecular Mechanics/Poisson−Boltzmann Surface Area) [35] methods, respectively.

### 2.5 ADMET analysis

The Shimadzu GC-MS-QP 2010 Ultra profiled substances were also subjected to toxicity evaluations using pkCSM [36]. The canonical SMILES for the molecular structure of each chemical were obtained from PubChem [37]. The compounds with the requisite physicochemical properties were subsequently investigated for their pharmacokinetic properties, as previously reported [38].

## 3 Results and discussion

### 3.1 PD-associated α-synuclein and flavonoid-based therapeutic potential

The production of unconventional, stable, and often disease-causing protein aggregates has more and more been attributed to a broad range of human disease. Of the numerous morphologies of protein aggregation, one structural type has particularly drawn attention from scientists. Interestingly, although unconnected amino acid sequences of varying constitution are able to form amyloid fibrils with varying chemical composition, the fibrils are invariably of extremely well-ordered structure [39]. This general architecture is characterized by anti-parallel or parallel β-sheet strands perpendicular to the long axis of the fibril, leading to so-called cross-β-sheet conformation [40]. Parkinson's disease (PD) is a prevalent neurodegenerative disorder, ranked as being the second most prevalent in the world after Alzheimer's disease (AD) [41]. Given its pre-eminent position within disease initiation and progression, α-synuclein is increasingly a front-runner among therapeutic intervention strategies for the control of PD progression [42]. It would thus be predicted that success with therapy would necessitate combinations of drugs acting on different pathological processes concomitantly. Although this multi-target approach has some limitations, e.g., pharmacokinetic problems with the adsorption, distribution, and elimination of drugs, and compromised compound permeability through the blood-brain barrier, the risk for toxic drug interactions might worsen CNS pathology. The ideal approach would be to identify single small molecules with multifunctional activity capable of modulating multiple age-dependent neurodegenerative processes within the brain [43]. Since there is now no effective treatment that targets the molecular basis of Parkinson's disease, there is a continuous search for new medications. Drug development methods for neurodegenerative diseases provide a rapid method for identifying drugs that have a high molecular target affinity and selectivity [44]. Because protein-based drugs have trouble passing across the Blood-Brain Barrier (BBB) and might cause collateral immunological reactions, tiny molecules are one of the preferred options for developing PD therapies [45]. Amongst the plant secondary metabolites, flavonoids are of special interest based on epidemiological suggestions towards their potential neuroprotective activity against neurodegenerative diseases. Polyphenolic compounds exhibit a preference for the attachment to the charged and natively disordered C-terminus of α-synuclein. This is observed in their capacity to degrade existing fibrils, reshape toxic aggregations, or to bias the generation of less toxic, off-pathway species [46]. In recent years, a number of natural bioactive molecules have been suggested as drugs against disabling neurodegenerative diseases with wide spectrums [47]. Based on current pathogenic theories, cytotoxic fibril deposition in a number of brain areas is indeed at the heart of these conditions. Therefore, ongoing discovery of molecules inhibiting or reversing protein aggregation is of high priority [48]. In this context, polyphenolic flavonoids have drawn growing scientific attention owing to their unique ability to form different types of molecular interactions [49,50]. Different computer simulations have added weight to the hypothesis that flavonoids ought to be able to control the new and dynamic path of disease-causing mutants [51,52]. Finally, recent experiments validate the therapeutic application of flavonoids in the prevention of the pathological oligomerization propensity of α-synuclein, a central etiopathogenic element of Parkinson's disease [53]. Some of these flavonoids include curcumin, baicalein, myricetin, epigallocatechin-3-gallate (EGCG) [29], and 7,8-dihydroxyflavone [54], and these have already been demonstrated to be anti-amyloid in diseases such as Parkinson's, Alzheimer's [55], transthyretin (ATTR) amyloidosis [22,56], and even potentially Fisetin and salvianolic acid A in SOD1-associated diseases [38,57].

In addition to their anti-amyloidogenic activity, these polyphenols display a diverse range of other biological activities, including enhancement of the prognosis for type 2 diabetes [58], antioxidant [59], antimutagenic activity, control of oxidative stress [60], anti-metabolic [61], modulation of apoptosis [62], anti-inflammatory activity [63], antimicrobial, and possible anti-cancer activity [64]. Computational simulation is used in this study to investigate the influence of natural polyphenols on the aggregation properties of α-synuclein, particularly in those areas most prone to the formation of neurotoxic amyloid conformations that are characteristic of Parkinson's disease.

### 3.2 Analysis of binding interaction

Molecular docking is a prominent computer-aided drug discovery technique that is used to forecast and score receptor-ligand interactions using binding energy. Molecular docking is among the primary methods for the prediction of protein target-ligand binding affinity [65]. In the current research study, extensive recent literature review was done with the objective of finding flavonoid molecules that possess therapeutic activity against amyloid-based neurodegenerative disorders.

**Fig. 1:**
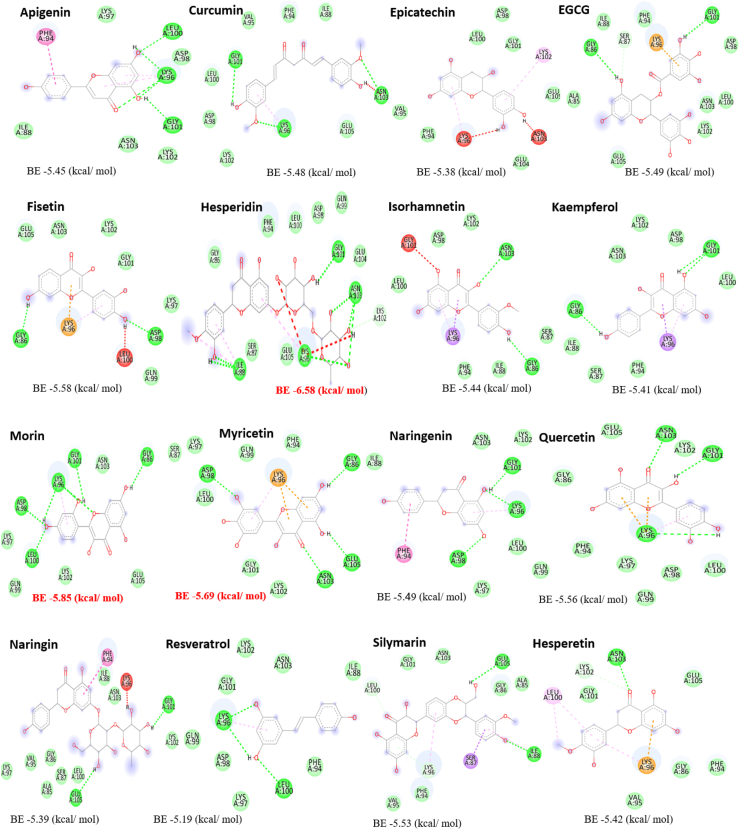
Flavonoids' docking binding energies with the α-synuclein protein were calculated. The chosen ligands' binding energy (kcal/mol) is indicated in red.

After careful consideration, 16 polyphenolic compounds that were known to possess the ability to disrupt amyloid fibril formation were chosen for preliminary computational screening against the amyloid-generating α-synuclein protein. These compounds were Apigenin, Epicatechin, Curcumin, Silymarin, Fisetin, Hesperetin, Hesperidin, Naringin, Myricetin, Morin, Isorhamnetin, Quercetin, Resveratrol, Naringenin, epigallocatechin gallate (EGCG), and Kaempferol [[66], [67], [68], [69]]. These flavonoids were categorized according to their likelihood of selectively binding with α-synuclein, and they were used for the first virtual screening round. Their binding energies against α-synuclein are shown in Fig. 1.

**Fig. 2:**
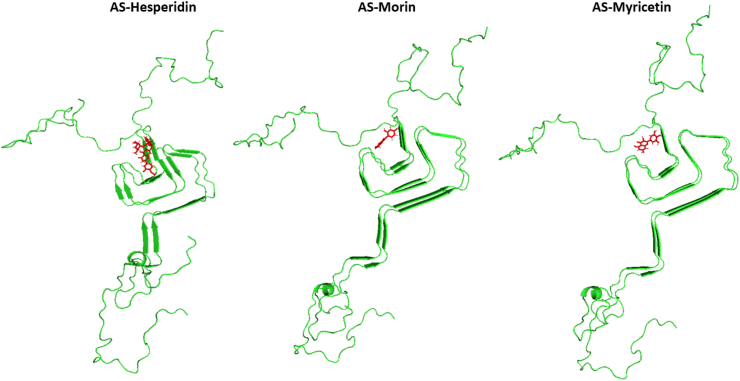
General schematic of the structure of α-synuclein protein and molecular docking performed on protein-ligands complexes by Hesperidin, Morin and Myricetin.

The more the drug or ligand exerts control over its bound target, the more effectively the drug and protein bond. The binding free energy is used to quantify the interactions between the ligand and the target. Hesperidin, Morin, and Myricetin were selected as potential substances for more investigation based on this criterion. Hesperidin was shown to have the best binding effectiveness with the α-synuclein protein, followed by myricetin and morin. As seen in Fig. 2, we selected the aggregation-prone areas as ligand-protein binding sites based on prior reports.

**Fig. 3:**
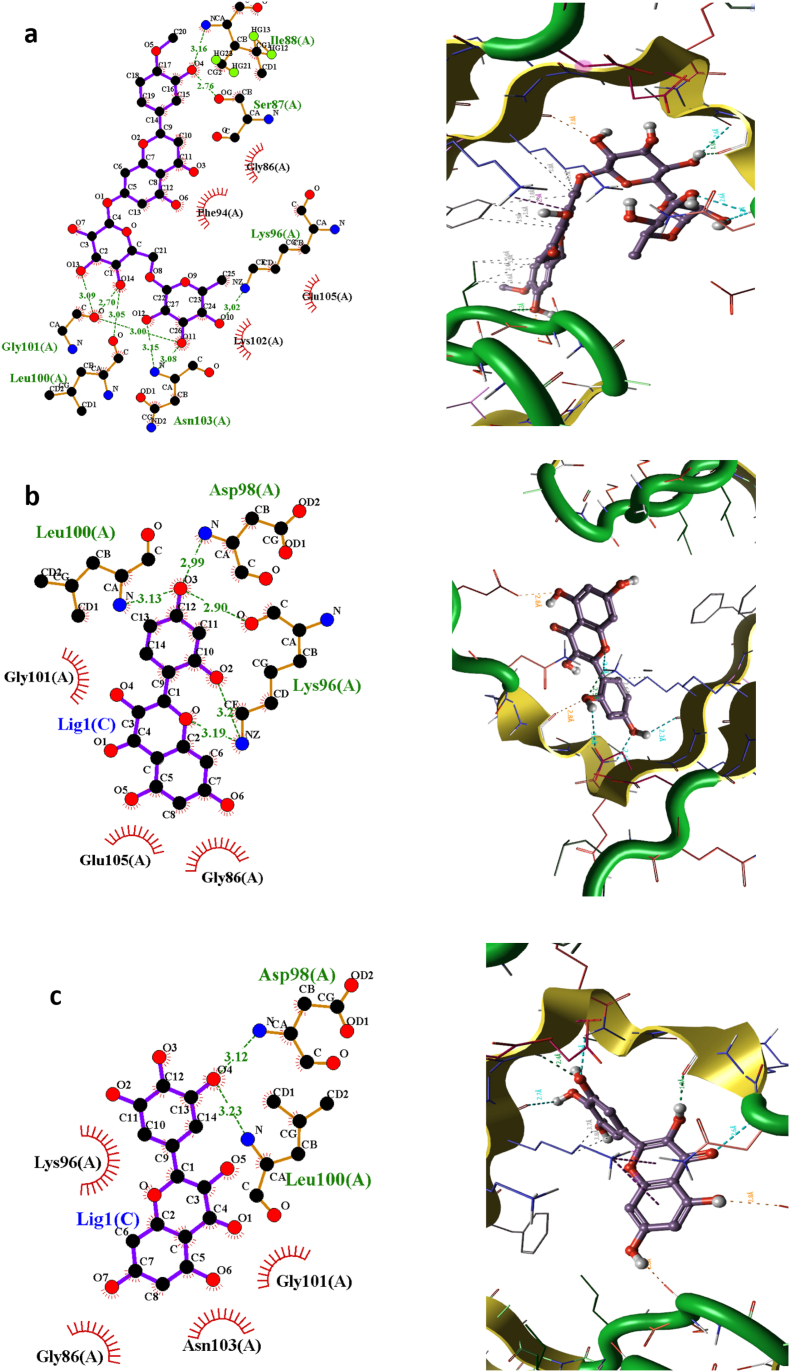
Visualization of variation in 2 and 3-D protein-ligand interactions between α-synuclein docked with a) Hesperidin, b) Morin and c) Myricetin.

The following are the binding locations in 3-D diagrams for each representative α-synuclein protein complex derived from cluster analysis:

The results demonstrated that Hesperidin bonded to the α-synuclein protein's Ser87, Ile88, Lys96, Leu100, Gly101, and Asn103 (Fig. 3a). With Lys96, Asp98, and Leu100, Morin established hydrogen bonds (Fig. 3b). Asp98 and Leu100 produced hydrogen bonds with myricetin (Fig. 3c). The increased hydrogen bond network in comparison to other flavonoid compounds was clearly caused by Hesperidin's changed molecular size and orientation in the α-synuclein protein, which resulted in the formation of six hydrogen bonds.

We also outlined the individual flavonoid binding sites for each flavonoid on the α-synuclein protein using two-dimensional interaction diagrams. Besides hydrogen bonding, dynamic intermolecular forces contribute significantly to tuning the stability and affinity of the protein-ligand complex. Interestingly, the aromatic rings of polyphenolic flavonoids are instrumental in creating hydrophobic contacts with α-synuclein complexes (see Table 1). Enhancement of such hydrophobic interactions is correlated with higher affinity of the ligand with the protein. More hydrophobic interaction at the active site of the complex is directly correlated with enhanced biological activity. Evidence from molecular docking studies provides support that flavonoids demonstrate strong interaction with α-synuclein capable of inhibiting or blocking its aggregation. Literature illustrates that hydrophobic interactions are the preferred mode for stabilizing ligand-protein affinity and enhancing the therapeutic efficacy of the compound [70]. In line with our molecular docking results, hesperidin, for example, has the capacity to destabilize protein aggregation through the establishment of beneficial contacts with α-synuclein and thus implying a strong anti-aggregation effect.

**Table 1:** Hydrogen and hydrophobic interactions formed by α-synuclein binding residues with protein-ligand complexes (Hesperidin, Morin and Myricetin).

protein–ligand complexes	Residues involving binding interactions	
	Hydrogen Bonds interaction	Hydrophobic interaction
α-synuclein-Hesperidin	Ser87, Ile88, Lys96, Leu100, Gly101 and Asn103	Gly86, Phe94, Lys102 and Glu105
α-synuclein-Morin	Lys96, Asp98 and Leu100	Gly86, Gly101 and Glu105
α-synuclein-Myricetin	Asp98 and Leu100	Gly86, Lys96, Gly101 and Asn103

### 3.3 Conformational changes in the geometry of AS and AS-protein-ligand complexes

**Fig. 4:**
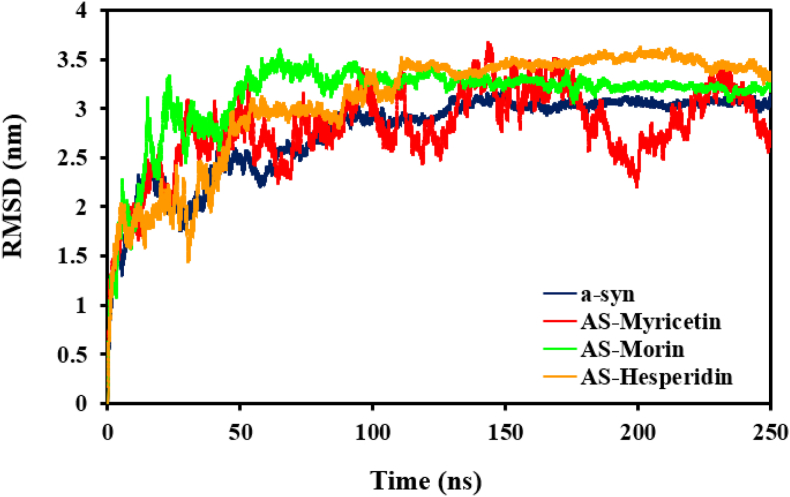
The α-syn protein's structural stability varies during the simulation period when it binds to the conformers of Myricetin, Hesperidin, and Morin.

To determine the conformational stability of α-synuclein Cα carbons and its flavonoid ligand complexes, we have calculated root mean square deviation (RMSD) values over the course of the simulation. The comparative plots are shown in Fig. 4. On the basis of RMSD analysis, it was seen that there is a clear difference in the trend of aggregated α-synuclein when alone compared with its flavonoid ligand complexes of hesperidin, morin, and myricetin. The free α-syn RMSD average was estimated to be around 2.75 ± 0.42 nm. Yet, the RMSD averages for the complexes of α-syn with Hesperidin, Morin, and Myricetin were 3.2 ± 0.39, 3.1 ± 0.57, and 2.81 ± 0.4 nm, respectively. Interestingly enough, structural variations of the α-syn protein were greatest in the complex with Hesperidin, implying that its binding causes considerable conformational changes. These findings show that Hesperidin destabilizes the native aggregation-prone α-syn structure more than the other flavonoids. Visual inspection of the trajectory of the simulation also confirms this, showing that the typical β-sheet structures—foreshadowing the rigidity of the protein—were destabilized during flavonoid interaction, most notably with Hesperidin. This demonstrates that ligand-induced loosening of structure is responsible for the reduction in conformational stability of α-synuclein in the complex with such polyphenolic compounds.

**Fig. 5:**
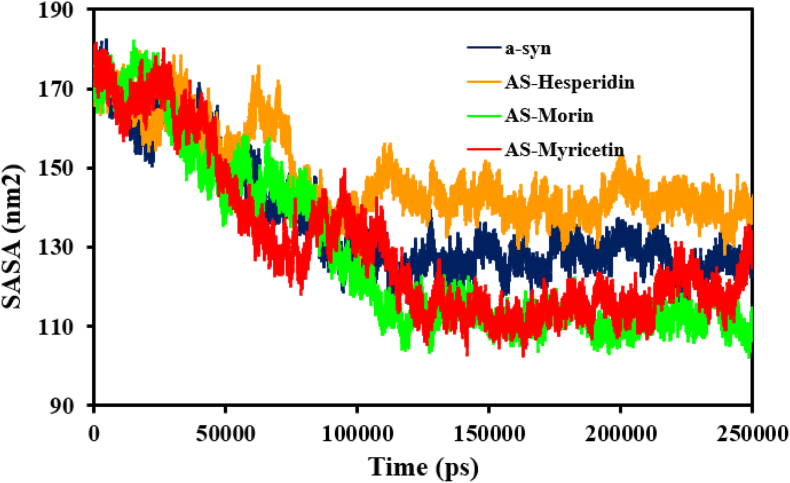
The solvent accessible surface area (SASA) of the α-syn protein during the simulation phase when it binds to the conformers of myricetin, hesperidin, and morin.

SASA, another significant structural metric, showed a similar pattern. SASA values were consistently greater than those of α-syn, with an average of 136 ± 15. nm^2^ for the α-syn protein and 147 ± 10, 128 ± 21 and 130 ± 19 nm^2^ for the α-syn protein–ligand complexes Hesperidin, Morin, and Myricetin (Fig. 5). According to these findings, hesperidin causes a looser conformation with chains that are easier to separate, exposing the underlying structure of aggregates, and disturbs α-syn more than other substances.

**Fig. 6:**
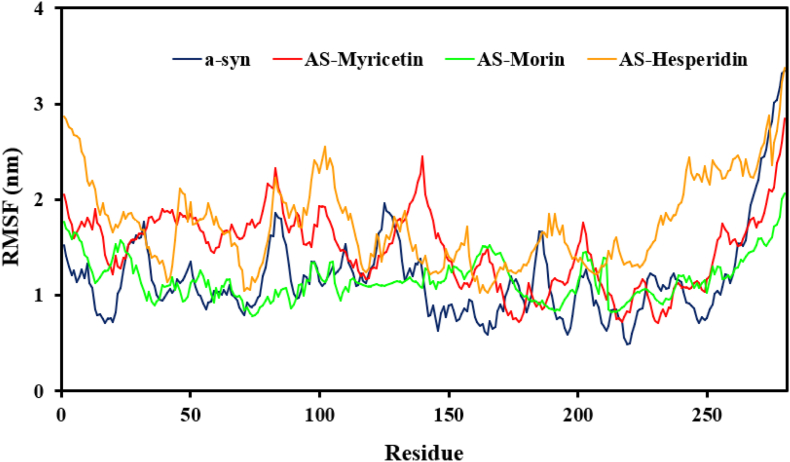
The early loss of residual flexibility upon interaction with myricetin, hesperidin, and morin is indicated by the conformational flexibility of the α-syn protein during the course of the simulation.

To develop a better insight into the structural behavior of the α-synuclein protein, we investigated its conformational flexibility using molecular dynamics (MD) simulations both flavonoid ligand-free and flavonoid ligand-bound (Fig. 6). Comparison revealed that complexation with such compounds profoundly affected the conformational motions of α-synuclein compared to its unbound conformation. RMSF was found to be 1.17 ± 0.45 nm for the free α-syn protein. With complexation with Morin and Myricetin, RMSF was raised to 1.16 ± 0.22 nm and 1.47 ± 0.39 nm, respectively. As seen earlier, highest increase was seen with Hesperidin, which raised the fluctuation to 1.74 ± 0.46 nm in α-*syn*-Hesperidin complex. These alterations are most probably due to particular interactions among the hydroxyl groups of flavonoid ligands and α-synuclein residues, disrupting the current intermolecular contacts and interfering with the protein structural stability and dynamics. Invariant tendencies in stability and flexibilities indices indicate that among all flavonoids tested, Hesperidin had the most profound effect on the protein conformations during the simulation. The significant reduction in the mobility of β-sheet structures of α-syn after ligand interaction corresponds to a general decrease in its conformational mobility, and thus flavonoid binding has to be stabilizing the protein against aggregation.

**Fig. 7:**
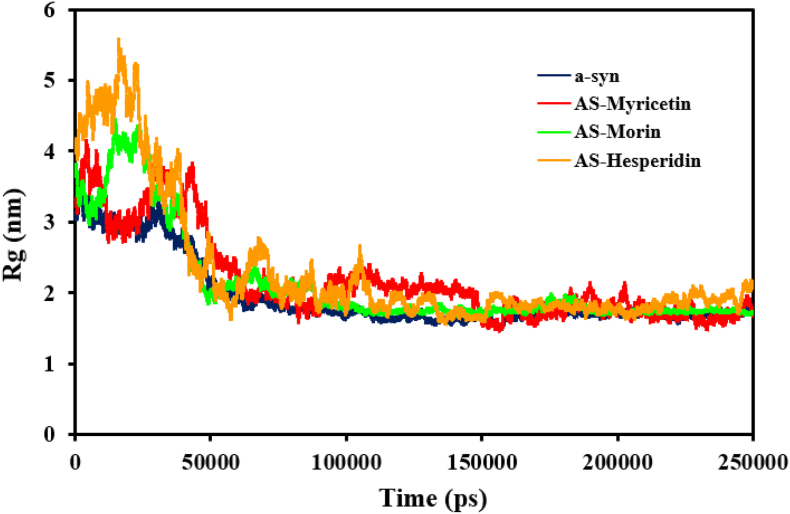
The function of protein compactness calculated for the α-syn protein during the simulation period when it bound to the conformers of myricetin, hesperidin, and morin. In contrast to α-syn protein, this suggests a general loss of compactness that subtly indicates a lack of intermolecular connections in protein–ligand complexes.

Second, the radius of gyration (Rg) was measured for α-synuclein in its complex with various polyphenolic flavonoids. Rg was calculated as the root mean square deviation of atomic positions from their collective center of mass, and it is a measure of overall protein structure compactness. α-Syn, in the free state, had an average Rg of 1.95 ± 0.48 nm. In complex with Hesperidin, the value increased slightly to 2.31 ± 0.91 nm in the course of the simulation. For the α-synuclein-Morin complex, the mean Rg shifted to 2.13 ± 0.68 nm, whereas interaction with Myricetin raised it to 2.18 ± 0.62 nm. Even these comparatively modest Rg deviations between the different complexes (Fig. 7) imply subtle but possibly relevant conformational effects. These results are consistent with the proposal that endogenous α-synuclein is in a more globular conformation than its flavonoid complexes. Structural characterization also concluded that this globular structure gets destabilized upon interaction of the protein with flavonoid molecules to adopt an open conformation. The initial data from MD simulations strongly suggest that flavonoids like Morin, Myricetin, and Hesperidin interfered with the native conformational structure of α-synuclein. The chemicals exhibited inhibitory effects on the protein's intermolecular interactions, altering its structural dynamics to result in aberrant pathological behavior. More research showed that Hesperidin interaction decreased intermolecular stabilization, indicating destabilizing action on the native structural equilibrium of α-syn.

**Fig. 8:**
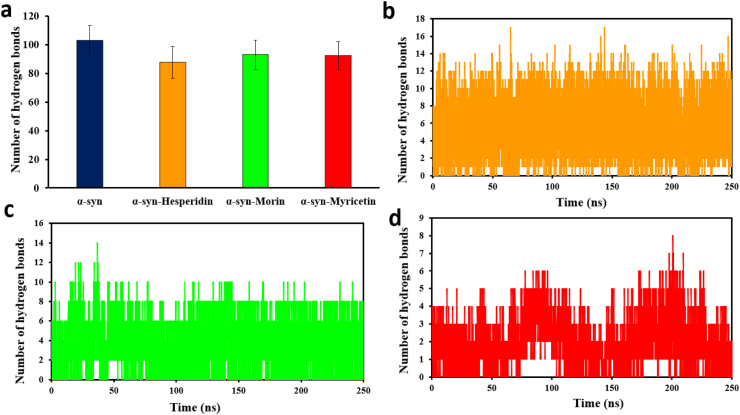
Examination of the hydrogen bonds that exist between α-syn and ligand complexes. (a) The total number of hydrogen bonds formed by ligand complexes and α-syn across the 250 ns MD simulation period. Plot illustrating the number of hydrogen bonds that were established between the α-syn and the conformers of b) Hesperidin, c) Morin, and d) Myricetin during the course of the simulation.

To compare the effect of flavonoid interactions on α-synuclein, we monitored hydrogen bond distances during molecular dynamics simulations with and without flavonoid ligand binding. Fig. 8a depicts the number of interpeptide hydrogen bonds between α-syn as a function of simulation time for 250 ns. The results indicated that flavonoid ligand binding resulted in the considerable reduction of hydrogen bonding between α-syn peptide chains relative to the unbound protein. In particular, unbound α-synuclein had an average of 103 ± 10 interpeptide hydrogen bonds. Myricetin-bound complex had an average of 92 ± 9, Hesperidin-bound complex 88 ± 11, and Morin-bound complex 93 ± 10 hydrogen bonds. Throughout the simulation, the number of hydrogen bonds between α-syn and each ligand was also monitored, averaging 2 ± 1 for Myricetin (Figs. 8d), 3 ± 1 for Morin (Figs. 8c), and 5 ± 2 for Hesperidin (Fig. 8b). These findings suggest that ligand binding profoundly changes the internal bonding environment of the α-synuclein molecule. The intense hydrophobic interactions between the protein and flavonoid ligands broke the native residue-residue contacts, reducing interpeptide cohesiveness and inducing structure rearrangement.

**Fig. 9:**
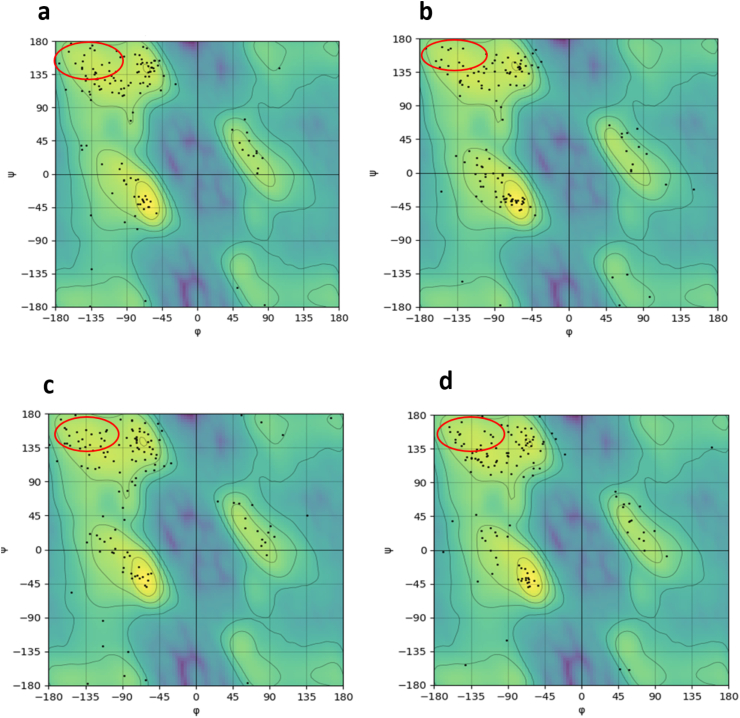
Depicts the secondary structure content Ramachandran plot for (a) the α-syn protein by itself and in combination with the flavonoids b) Hesperidin, c) Morin, and d) Myricetin.

As confirmed by secondary structure analysis (Fig. 9) and earlier structural integrity determinations (Fig. 4), we conclude that the flavonoid ligands disrupted native hydrogen bonding, functionally depolymerized the primary assembly, and disrupted the stability of α-syn's interpeptide framework.

The formation of secondary structural motifs—mostly β-sheets—is one of the important aspects in the context of protein aggregation present in most neurodegenerative conditions. α-Synuclein aggregates are mostly composed of β-sheet structure, an aspect well-reported in experimental and computational literature [71,72]. Experimental data also authenticate that amyloidogenic proteins have high β-sheet composition, which would be a universal point of interest in pathogenesis and disease state development [73]. In this, we have determined secondary structure changes of α-synuclein during the course of molecular dynamics simulation with the assistance of the DSSP (Define Secondary Structure of Proteins) algorithm, which monitors the change in secondary structural components as a function of time. These are presented in Table 2 and indicate how secondary structure fingerprint of protein and its complexes have evolved during simulation time. To gain clearer insights into the conformational dynamics, especially β-sheet structures and their implications on protein autophagy functions, we generated Ramachandran plots for both the mutant α-synuclein as well as its liganded forms (Fig. 8). These are Python analysis software-generated plots that present the distribution of phi (Φ) and psi (ψ) dihedral angles for all residues and provide information on stereochemical validity and folding propensities of the protein structures under various conditions.

**Table 2:** Secondary structural analysis DSSP (Define Secondary Structure of Proteins) algorithm related to α-syn protein and its flavonoid-based compounds (Hesperidin, Morin and Myricetin).

Secondary structure elements (%)	Coil	β-Sheet	β-Bridge	Bend	Turn	α-Helix
α-*syn* protein	28	35	8	11	13	5
α-*syn*-Hesperidin	32	29	7	12	11	8
α-*syn*-Morin	31	33	7	12	10	7
α-*syn*–Myricetin	32	31	6	13	11	7

The secondary structural component analysis revealed that the native α-synuclein had a very high number of β-sheets, around 35 %, as could be inferred from Fig. 9a. Upon binding of hesperidin to it, there was a strong decrease in the β-sheet formation, with the content dropping to 29 %. This is evidence of the strong disruption of the β-sheet structure upon flavonoid binding. The results from RMSD and SASA analyses also assure us that protein-ligand complexes cause substantial changes in the predominant beta-sheet conformation of native α-syn. Structural perturbations are emphasized by local differences in the β-sheet areas, depicted as yellow circles in Fig. 9b. Consistently, the β-sheet content dropped to 33 % upon addition of Morin (Fig. 9c) and to 31 % with Myricetin (Fig. 9d), demonstrating that the three flavonoids affect the β-sheet structure to some extent. Of these, Hesperidin had the maximum effect. The significant increase in random coil content of these complexes indicates destabilization of ordered β-sheet conformation, suggesting movement towards less aggregated and disordered conformation. Reorganization of this secondary structure attests that loss of β-sheet components is a primary contributor to increased occurrence of other structural motifs within protein-ligand complexes. Overall, these findings indicate that the interactions between α-syn and flavonoid compounds like Hesperidin have the capacity to disrupt the amyloidogenic β-sheet core, thereby inhibiting aggregation pathways. This identifies Hesperidin as a potent inhibitor of α-synuclein amyloidogenesis. Additionally, the results of the secondary structural propensities are consistent with studies that have been reported before [74].

### 3.4 Principal component and free-energy landscape analysis

All macromolecules move in a useful way. Their atomic motions have complex interactions that result in them. Its proper functioning is dependent on these internal motions, including structural changes in different living conditions. Understanding many of the intrinsic dynamics of protein molecules is challenging. Principle component analysis, often known as PCA, is the technique of dissecting big data sets into their primary components and determining the major changes that most accurately depict the global protein movement with important details.

**Fig. 10:**
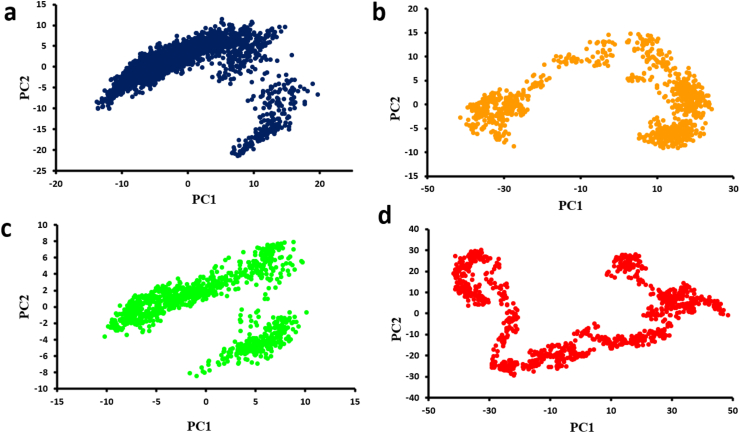
Plot plots of the mean principal component analysis of a) α-syn, b) Hesperidin, c) Morin and d) Myricetin, exploration of conformational and dynamical space during 250 ns simulations.

Each PC derived from MD simulations, which are eigenvector values obtained from the covariance matrix, effectively represents a change in the protein trajectory. The first step was to extract the eigenvector values from the simulated covariance matrix. The following determines the motions of the main protein after filtering many trajectories [75]. A two-dimensional (2D) PCA was performed to investigate the dynamics of protein/ligand systems and understand how ligand binding influences protein movements. Because PCA analysis greatly lowers the high dimensionality of crucial variables, including atomic coordinates and dihedral angles, in the simulation, conformational changes in the α-syn protein were carried out using this method. The trajectory projection map Fig. 10 shows that all system locations are centered around the origin, suggesting that all systems oscillate around an equilibrium state during the simulations. Furthermore, across all ligand-bound forms, the apoprotein exhibited the biggest PCA plot, whereas the α-syn protein's PCA plots revealed tighter findings for all ligands. These findings imply that, in the absence of a ligand, the protein can explore a greater range of structural states due to its inherent flexibility. Conversely, the Rg and SASA graphs indicate that the introduction of ligands leads to the protein complexes becoming denser and more clustered. Fig. 10 displays the projections of the Cα trajectory onto the eigenvectors (vectors 1 and 2) obtained from the covariance matrix across different time intervals. The matrix was diagonalized by utilizing a single trajectory of both the bound and unbound α-syn proteins. After diagonalization, the trace of the unbound α-syn covariance matrix indicated reduced movement amplitudes as the covariance matrix expanded. A trace of the covariance matrix in the bound state (Hesperidin) revealed distinct, grouped movements that occupied less space as the covariance matrix decreased (Fig. 10b). This flavonoid's morin binding resulted in more significant protein structural alterations that affected hydrogen bonds more, causing the PC1PC2 plot to display more dispersed spots (Fig. 10c). The scatter plot findings for myricetin binding show that this flavonoid altered the structure of the protein. PC1PC2's linked and integrated pathways result in fewer modifications to the protein's secondary structure and restricted alterations to its spatial layout (Fig. 10d). The protein may become more dynamically unstable as a result of this dispersion. Plot alterations in the presence of Hesperidin are comparable to those generated by Myricetin, but there is a little more dispersion, suggesting that Hesperidin has significantly altered the protein structure and that its effects are more pronounced than Morin's.

**Fig. 11:**
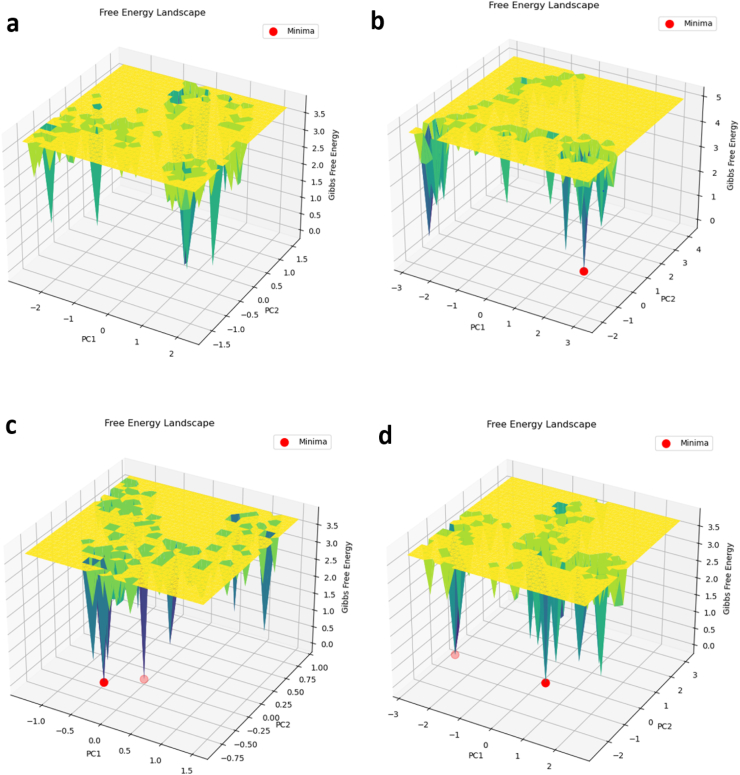
Studying the pathways of a) α-syn and b) changes in the α-syn protein following contact with b) hesperidin, c) morin, and d) myricetin yielded the free energy landscape (FEL).

Protein conformational space is more accurately represented in terms of energy and time using PCA-based free energy landscape (FEL) analysis. FEL was evaluated in order to understand the conformational changes of protein-ligand complexes and α-syn. The FEL represents the total number of interactions and residue-to-residue connections that correspond to the most stable native structure. The propensity of amyloids to be polymorphic is one of their main traits, and FEL was interpreted to further investigate several aspects of amyloid pathogenesis from this perspective [76]. The link between structural states affected by the free energy landscape must be shown in order to identify the cause of protein aggregation. To compute the FEL, GROMACS analysis modules were used. A Comparable FEL contour map was produced by projecting the first (PC1) and second (PC2) eigenvectors; deeper blue colors indicate lower energy levels. Fig. 10 suggests that the aggregates of the α-syn protein have reached several energy minima that clash with metastable states during the course of conformational time. The overall increase in the number of conformers that unfold the global free energy minimum in α-syn further supported the protein's larger range of dispersion. Thus, the result indicates that the increasing percentage of many conformers with global energy minimum in α-syn has approaching the fully unfolded states. The unfolded state has produced maximum conformations in α-syn with Gibbs free energy ranging from 0 to 3.5 kcal/mol (Fig. 11a). In conclusion, our findings suggest that irreversible changes in the α-syn conformational configurations promote the creation of dangerous aggregates in the protein. Previous studies that showed stacked proteins achieve several global energy minima for conformations also corroborate our findings. Conversely, it is anticipated that the binding of Hesperidin with α-syn will be limited to a specific funnel with lower values on the free energy landscape. Additionally, the conformers fall within the Gibbs free energy range of 0–5 kcal/mol (Fig. 11b). Because Hesperidin binding more dramatically reduced the dispersion of the combined structures, the global free energy minima of the α-*syn*-Hesperidin conformers were confined to a specific region [77]. In conclusion, our examination of the free energy profile demonstrated that the α-syn protein's capacity to form stacking conformers was reduced upon contact with Hesperidin. Therefore, it was crucial to determine how Hesperidin influenced the formation of aggregates in the α-syn conformers in order to reach their nearest global energy minimum. Thus, it is clear from the findings of our investigation that hesperidin reduces the cytotoxicity that the misfolded aggregates of α-syn protein create by preventing their formation. When Morin and Myricetin are present, the free energy is reduced (Fig. 11c and d), indicating that the protein structure is more stable. The energy minima emerge as sharp, scattered dots, suggesting better stability and less structural changes. Hesperidin enhanced instability by altering secondary protein structure and hydrogen bonding more than Morin did. Overall, the tests of molecular docking, geometric evaluations, secondary structure propensity, and free energy perspective showed that Hesperidin had an inhibitory effect on aggregated α-syn protein.

### 3.5 Molecular mechanics Poisson Boltzmann surface area calculations

The MM-PBSA method is commonly viewed as an extremely efficient and computationally economical way of approximating ligand binding affinities to proteins [35]. One of the most widely used methods for approximating interaction energies, MM-PBSA achieves a satisfying balance between accuracy and time [78]. Table 3 shows the calculated binding free energies and their constituent terms for a representative subset of α-synuclein–flavonoid complexes. The interaction energies of the α-synuclein complexes with Hesperidin, Morin, and Myricetin were calculated to be - −92.69 ± 0.31, −83.95 ± 2.77 and −77.54 ± 2.86 kJ mol^−1^, respectively. The negativity of the values suggests that binding is favorable and verifies that all three flavonoid compounds directly interact with the α-synuclein protein. Hesperidin, in particular, also had the greatest affinity among the compounds tested, as seen in earlier molecular docking results and substantiating it as a lead compound. The values for higher binding affinity are generally associated with higher capacity for inhibiting protein aggregation—a key aspect of neurodegenerative disease treatment. Additional breakdown of the total binding energy showed that nonpolar contacts (van der Waals interactions and nonpolar solvation contributions) and electrostatic terms (electrostatic energy and solvent-accessible surface area or SASA) were the primary positive factors. Polar solvation energy, however, always opposed the total binding energy in all the complexes under investigation. According to these findings, Hesperidin was a potential candidate for modulating the α-synuclein aggregation pathway. By stabilizing protein structure and blocking pathological conformational shifts, it can act as an effective therapeutic flavonoid against Parkinson's disease.

**Table 3:** The values of the free energy components of binding between different α-syn protein complexes include van der Waals energy, Electrostatic energy, Polar solvation, SASA energy, and Total binding energy.

Energy components (kJ mol^−1^)	Hesperidin	Morin	Myricetin
Van der Waals	−216.35 ± 4.18	−270.87 ± 6.05	−205.32 ± 7.39
Electrostatic	−82.56 ± 7.14	−93.04 ± 9.93	−62.86 ± 1.48
Polar solvation	232.73 ± 12.79	311.87 ± 6.34	219.12 ± 4.38
SASA energy	−26.51 ± 1.21	−31.91 ± 0.34	−28.47 ± 1.34
ΔE binding	−92.69 ± 0.31	−83.95 ± 2.77	−77.54 ± 2.86

### 3.6 In silico drug ADMET evaluation

New drug development is becoming more difficult, costly, risky, and less successful every time. Pharmacokinetics, toxicity, and potency must all work together for drugs to be effective. The creation of computer methods to optimize toxicity and pharmacokinetic properties may expedite and enhance the search for novel drug candidates. It is difficult to predict ADMET-related properties of new drugs since pharmacokinetic and toxicological properties are not well connected with a number of physicochemical properties. There is a need for innovative approaches to understand, research, and predict the ADMET properties of tiny compounds in order to improve compound quality and success rate [79]. The logical creation of anti-PD medications depends on a drug's pharmacokinetic characteristics. As a result, the test chemicals' toxicity was assessed in silico. Table 4 presents the findings. Three compounds were examined utilizing the pKCSM technique in ADMET screening. The LD50, *Salmonella typhimurium*/reverse mutation assay (AMES assay), and hepatotoxicity prediction provide the basis for the toxicity potential parameter [80]. It was also anticipated what the human maximum acceptable dosage would be. These results correspond with the ADMET properties of flavonoids that have been earlier studied to understand their pharmacokinetic profile [81].

**Table 4:** Prediction of Absorption, Distribution, Metabolism, Excretion and Toxicity (ADMET) parameters of Hesperidin Morin Myricetin compounds using the pKCSM program.

Parameter		Hesperidin	Morin	Myricetin	Unit
Absorption	Water solubility	−3.014	−2.978	−2.915	(log mol/L)
	Caco2 permeability	0.505	−0.294	0.095	(log Papp in 10 ^−6^ cm/s)
	Intestinal absorption (human)	31.481	75.408	65.93	(% Absorbed)
	Skin Permeability	−2.735	−2.735	−2.735	(log Kp)
	P-glycoprotein substrate	Yes	Yes	Yes	Categorical
	P-glycoprotein I inhibitor	No	No	No	Categorical
	P-glycoprotein II inhibitor	No	No	No	Categorical
Distribution	VDss (human)	0.996	1.229	1.317	(log L/kg)
	Fraction unbound (human)	0.101	0.214	0.238	(Fu)
	BBB permeability	−1.715	−1.18	−1.493	Categorical (log PS)
	CNS permeability	−4.807	−3.389	−3.709	
Metabolism	CYP2D6 substrate	No	No	No	Categorical
	CYP3A4 substrate	No	No	No	Categorical
	CYP1A2 inhibitior	No	Yes	Yes	Categorical
	CYP2C19 inhibitior	No	No	No	Categorical
	CYP2C9 inhibitior	No	No	No	Categorical
	CYP2D6 inhibitior	No	No	No	Categorical
	CYP3A4 inhibitior	No	No	No	Categorical
Excretion	Total Clearance	0.211	0.486	0.422	(log ml/min/kg)
	Renal OCT2 substrate	No	No	No	Categorical
Toxicity	AMES toxicity	No	No	No	Categorical
	Max. tolerated dose (human)	0.525	0.537	0.51	Log mg/kg/day)
	hERG I inhibitor	No	No	No	Categorical
	hERG II inhibitor	Yes	No	No	Categorical
	Oral Rat Acute Toxicity (LD50)	2.506	2.413	2.497	(mol/kg)
	Oral Rat Chronic Toxicity (LOAEL)	3.167	2.448	2.718	(Log mg/kg_bw/day)
	Hepatotoxicity	No	No	No	Categorical
	Skin Sensitization	No	No	No	Categorical
	*T.Pyriformis* toxicity	0.285	0.308	0.286	Log ug/L)
	Minnow toxicity	7.131	4.684	5.023	(Log mM)

Hesperidin (Hesp; molecular formula: C_28_H_34_O_15_; IUPAC name: 3′,5,7-trihydroxy-4′-methoxy-flavanone-7-*O*-rutinoside) is a flavanone glycoside known for its limited solubility in water and many organic solvents [82]. Belonging to the polyphenolic flavonoid family—more specifically, to the flavanone subclass—hesperidin is structurally based on a three-ring diphenylpropane backbone (C6–C3–C6) [83]. Flavanones are chemically defined by a carbonyl group at the C4 position and the absence of a C2–C3 double bond in the central C-ring [84]. Flavonoid glycosides are the most common form of flavonoids present in nature, typically featuring O-glycosidic bonds involving the hydroxyl groups on the flavonoid structure. Some variations include C-glycosides, where sugar moieties are covalently bonded through carbon-carbon linkages. Chemically, hesperidin consists of two main components: a flavonoid aglycone, hesperetin, which is 3′,5,7-trihydroxy-4′-methoxyflavanone, and a sugar component—rutinoside (a disaccharide of rhamnose and glucose)—attached to the C7 position of the flavonoid core. Hesperidin has a molecular weight of 610.57 Da [85]. Citrus fruits are especially rich in hesperidin. It is abundantly present in sweet oranges (Citrus sinensis), lemons (Citrus limon), bitter orange (Citrus aurantium), citron (Citrus medica) [86], clementines (Citrus clementina) and mandarins (Citrus reticulata) [87]. Additionally, it is found in certain herbs such as peppermint (Menthae piperitae), St. John's wort (*Hypericum perforatum*), and sage (Salvia officinalis) [87]. In pharmacological evaluations, bioavailability is a key parameter—especially for orally administered drugs, which remain the most convenient and prevalent form of treatment for many chronic and acute conditions. Among the several determinants of oral bioavailability, solubility and membrane permeability are particularly critical [88,89].Encapsulation, which permits regulated release over time and, in most circumstances, enhances the solubility of molecules in water, can also help Hesp and some of its derivatives with their poor bioavailability. Emulsification techniques include extrusion, self-assembly, freeze-drying, and spray-drying, albeit the eventual use greatly influences the encapsulation method selected. The most prevalent carrier materials are polysaccharides, which are followed by proteins, lipids, oligosaccharides, and glycoproteins [82]. Given their high life expectancy, neurodegenerative disorders are expected to become a significant issue in the years to come. Given that many chronic illnesses are age-related, the older the population, the more likely it is that disorders linked to a decline in brain function will be problematic for health services. Two characteristics of neurodegenerative disorders are (i) the susceptibility of certain brain areas' neurons to disease, and (ii) the disorder's development and deterioration with time. This group of illnesses includes ailments including Huntington's disease, Parkinson's disease, and Alzheimer's disease [90]. Numerous possible mechanisms of action emerge due to the complex biology of neurodegenerative disorders. Hesperidin appears to have the potential to develop into a viable neuroprotective drug because of its anti-inflammatory, anti-amyloidogenic, anti-apoptotic, and antioxidant properties. The following studies show that hesperidin has neuroprotective properties:

Alzheimer's disease (AD) has been demonstrated to benefit neuroprotectively from hesperidin. In the APP/PS1–21 mice model of AD, it was demonstrated to decrease TGF-β, microglial activation, and β-amyloid formation [91]. Furthermore, it was observed that hesperidin increased GSH, mitochondrial complex I–IV activity, and total antioxidant capacity in the APPswe/PS1dE9 transgenic mice model of AD while lowering MDA and hydrogen peroxide levels with GSK-3β [92]. According to another study, aluminum chloride produced Alzheimer's in rats, and HPD decreased levels of acetyl cholinesterase, amyloid precursor protein (APP), Aβ1–40 deposition, and β and γ secretase [93]. Hesperidin neuroprotective action was triggered in sporadic Alzheimer's-type dementia caused by intracerebroventricular streptozotocin by lowering AChE and TBARS and decreasing NF-κB, COX-2, and iNOS as ganglioside levels increased [94]. It has been shown that l-methionine causes hyper-homocysteinemia in rats with vascular dementia, while hesperidin has a protective effect by raising CAT, GSH, and SOD while lowering AChE, MDA, serum nitrite, and serum homocysteinemia (Hcy) [95,96]. Research has demonstrated that hesperidin inhibits the progression of neurological illnesses, such as multiple sclerosis, Parkinson's, Alzheimer's, Huntington's, and amyotrophic lateral sclerosis [97]. Hesperidin may be a possible lead molecule for the development of mutant SOD1 inhibitors for the treatment of ALS, according to study data [98]. According to recent research, forsythoside B and echinacoside make α-Syn more fluid in condensates, stop it from transferring into amyloid fibrils, and lessen the toxicity that fibrils cause in SH-SY5Y cells [99]. Additionally, the findings demonstrated that carefully crafted peptides that targeted α-/β-synuclein interaction sites prevented α-synuclein's liquid-solid phase separation (LSPS), restored locomotor abnormalities, and increased the longevity of the *Caenorhabditis elegans* strain NL5901 [100].

The aim of this study was to find out which of the three flavonoid compounds—hesperidin, morin, or myricetin—is the most therapeutically potent in inhibiting the disease-induced effects caused due to α-synuclein (α-syn) aggregation. A composite analysis was made based on previous assessments of flavonoid binding ability, impact on β-sheet structural formation, and conformational dynamic change of α-syn. Among the compounds screened, hesperidin was the strongest in inhibiting the amyloidogenic activity of α-syn and therefore the most capable of neutralizing neurotoxic protein aggregation. Conversely, whereas from our results, neither myricetin nor morin—despite being highly documented polyphenolic flavonoids with documented capability to neutralize amyloidogenic proteins such as SOD1, amyloid-beta, and huntingtin—performed optimally in inhibiting α-syn aggregation from this study [67]. But it needs to be kept in mind that there is no experimental evidence for such flavonoid-protein interactions. Proper criticism of previously reported literature with α-syn and other flavonoids serves to instill a strong connection between theoretical models and experimental evidence towards these molecules' neuroprotection [[101], [102], [103]]. Although our computational results always gravitated towards hesperidin, our measured interactions also place morin as a valuable alternative of potential interest in preventing the toxic action of α-syn. These results can be useful to current and future studies in the design of morin-derived therapeutic compounds designed to fight against Parkinson's disease by interrupting α-syn aggregation processes.

## 4 Conclusions

Natural product-derived small molecule inhibitors have also persisted with vast therapeutic possibilities against various neurodegenerative disorders. Current pharmacological advancements identify flavonoids to be of value against their anti-amyloidogenic action, especially in blocking pathological proteins like misfolded α-synuclein. In this study, we utilized computational analysis—molecular docking and molecular dynamics simulation—to examine the interactions of three compounds of flavonoids (hesperidin, morin, and myricetin) with the α-syn dimer. We report a flavonoid-mediated therapeutic paradigm with the capacity to prevent effectively the pathological conformational transformation of α-syn into amyloid form. While experimental evidence is still limited, simulation-based assessment and invariably positive ligand-protein interaction results reaffirm hesperidin's immense therapeutic potential. The findings render hesperidin an exceptionally promising compound to be further developed as an α-syn aggregation modulator. Our research hence brings about a valuable step towards structure-based drug discovery initiative against neurodegeneration, in favor of the therapeutic utility of natural polyphenolic molecules towards treating Parkinson's disease.

## Consent to publish

The authors confirm that informed consent was given by human study participants for publishing.

## CRediT authorship contribution statement

Zainab Abdullah Waheed and Haider Khabt Aboud contributed to methodology, data curation, formal analysis, writing—original draft preparation. Jasem Hanoon Hashim Al-Awadi data curation, formal analysis. Azhaar Mousa Jaffar Al-Mousawy and khudhair Rashid khudhair Alzubaidi Project administration, formal analysis, data curation, contributed to the methodology, writing—review and editing, data curation, Software.

## Funding

The authors declare that no funds, grants, or other support were received during the preparation of this manuscript.

All of the study's individual subjects gave their informed consent.

## Declaration of competing interest

The authors declare that they have no known competing financial interests or personal relationships that could have appeared to influence the work reported in this paper.

## Data Availability

Data will be made available on request.
